# Monitoring and evaluation in disaster management courses: a scoping review

**DOI:** 10.1186/s12909-025-06659-0

**Published:** 2025-02-06

**Authors:** George Teo Voicescu, Hamdi Lamine, Andra Elena Loșonți, Eugenia Maria Lupan-Mureșan, Sonia Luka, José García Ulerio, Luca Ragazzoni, Francesco Della Corte, Marta Caviglia

**Affiliations:** 1 CRIMEDIM—Center for Research and Training in Disaster Medicine, Humanitarian Aid and Global Health, Università del Piemonte Orientale, 28100 Novara, Italy; 2 Emergency Medicine Discipline - Department 6 Surgery, “Iuliu Hatieganu” University of Medicine and Pharmacy, Cluj-Napoca, Romania; 3 Department for Sustainable Development and Ecological Transition, Università del Piemonte Orientale, Vercelli, Italy; 4 Department of Community Health Nursing, College of Nursing, University of Hail, Hail, Saudi Arabia; 5 Department Paediatrics III, Emergency Clinic Hospital for Children, Cluj-Napoca, Romania; 6 Emergency Medicine Department, Emergency Clinical County Hospital Cluj, Cluj-Napoca, Romania; 7 Department of Translational Medicine, Università del Piemonte Orientale, Novara, Italy

**Keywords:** Capacity Building standards, Educational Measurement, Disaster Medicine education, Disaster Planning, Monitoring and Evaluation, Disaster Management

## Abstract

**Background:**

Owing to the infrequent emergence of disasters and the challenges associated with their management, responders need appropriate training beyond doubt. Ensuring the highest standard of disaster management (DM) training is of paramount importance for high-quality DM. However, the literature concerning DM training monitoring and evaluation (M&E) is scarce. The primary objective of this review was to document the existing M&E strategies for DM training.

**Methods:**

The authors conducted a systematic literature search on June 28, 2023, on the PubMed, Scopus, Embase and Cochrane databases, including studies that described the learning objectives and the M&E strategy of DM training. The authors categorized the learning objectives and the evaluation methodology according to the revised Bloom’s Taxonomy and the New World Kirkpatrick model, respectively.

**Results:**

Fifty-seven articles met the inclusion and exclusion criteria, described DM training targeting healthcare and non-healthcare professionals and employed diverse teaching methods and topics. Five studies reported using monitoring, while all reported an evaluation methodology. The learning objectives focused on students’ ability to “Remember” (*N* = 50) and “Apply”(*N* = 44). The evaluations centred around the second level of the New World Kirkpatrick model (*N* = 57), with only 7 articles investigating the third level. Sixteen authors used existing, validated M&E frameworks. When corelating the learning objectives with the evaluation methodology, the authors observed a mismatch, as skills like the students’ ability to “Apply” and “Create” were evaluated using the second level of the New World Kirkpatrick model.

**Conclusions:**

The great heterogeneity in DM training highlights the particularity of these educational programs. The lack of monitoring and the low usage of existing M&E frameworks highlighted a lack of awareness and standardization in the field. The mismatch between the learning objectives and the evaluation process led to deceptive evaluations, which may have resulted in graduates being deemed ready to deploy despite facing hardships in real-world settings, potentially leading to unprepared responders.

**Supplementary Information:**

The online version contains supplementary material available at 10.1186/s12909-025-06659-0.

## Background

Disaster management (DM), as defined by the World Health Organization (WHO), involves the systematic coordination, planning, and execution of strategies designed to prepare for, respond to, and recover from catastrophic events [1]. Due to the infrequent emergence of disasters and the challenges associated with their management, assessing the performance of those responding to these events is very difficult. Therefore, to ensure responders’ preparedness, it is essential to verify that they have been trained effectively. The importance of training in the field of DM has been widely emphasized, with numerous calls for its improvement [2, 3]. Several after action reports highlighted that effective DM requires efficient training, which not only benefits the affected population but also increases the willingness and confidence of professionals in performing their duties [4, 5].

In 2002, the United Nations recognized monitoring and evaluation (M&E) as a key element for enhancing learning and promoting education [6]. While monitoring represents a continuous process aimed at providing timely data on training progress to allow organizers to correct deficiencies early, evaluation constitutes an episodic, objective examination focused on assessing training efficiency, impact, and effectiveness [7]. Among the most common types of monitoring are process, compliance, context, financial and beneficiary monitoring, while formative, summative, midterm, final or ex-post evaluation represent some of the most widely used evaluation strategies [8].

Regrettably, despite the increasing number of training programs developed over the years, the standardization and consistency of introducing M&E approaches have been lacking and rather arbitrary [9–13]. The importance of standardized quality management systems and tools for evaluating learning is also emphasized by the WHO’s learning strategy [14]. However, in the field of DM education and training, it remains unclear how extensively M&E approaches are implemented and whether they align with the learning objectives set by trainers.

The literature on the DM core competencies is widely heterogenic, as they range from situation awareness, communication, personal safety measures to surge capacity principles, public health management, ethics and legal principles [15, 16]. The diversity and complexity inherent in DM education and training pose significant challenges for their M&E [9, 17]. Indeed, DM training programs currently encompass a wide range of topics, ranging from disaster medical response and patient management to planning and coordination strategies, and utilize various teaching methods, such as e-learning, traditional classroom lectures, and multiple types of simulations [10].

Therefore, the primary objective of this review was to identify the existing M&E strategies for DM training. The secondary objective was to analyse the relationships between the learning objectives and the evaluation methods (Table 1).

**Table 1:** PICOT framework for the systematic scoping review [18]

Population	Healthcare and non-healthcare workers trained or currently in training in disaster management
Interest	Monitoring and evaluation approaches, tools and/or frameworks used in disaster management courses
Context	Courses on the topic of disaster management, regardless the level of study or delivery settings
Outcome	Document the monitoring and evaluation approaches, frameworks and tools used in disaster management
Types of study	Primary studies design of peer-reviewed and non-peer-reviewed journals

## Methods

The present scoping review followed the Preferred Reporting Items for Systematic Reviews and Meta-Analyses extension for Scoping Reviews (PRISMA-ScR) [19]. Prior to the literature search, the authors developed a review protocol according to the recommendations of Peters et al. [20, 21].

A systematic literature search was conducted on June 28, 2023, on PubMed, Scopus, Embase and Cochrane databases. The search strings were created in collaboration with a professional librarian, combining three different search terms: “disaster”, “training”, and “evaluation/monitoring”.

The identified articles were uploaded into the Rayyan Intelligent Systematic Review tool, and duplicates were automatically removed [22]. Title and abstract screenings were independently conducted by two researchers in accordance with predefined inclusion criteria. Following this initial phase, articles meeting the inclusion criteria underwent full-text examination. In instances where full-text versions were unavailable, efforts were made to establish communication with corresponding authors. Any screening outcomes were reconciled through mutual consensus at the conclusion of each phase.

### Inclusion and exclusion criteria

Articles that described a monitoring and/or evaluation approach/tool/framework used for disaster management courses aimed at disaster management professional workers were included. Disaster management professionals consider all the skilled workers in any of the fields encompassed in the WHO definition of “Disaster Management”, including those that are currently in training (e.g. undergraduates) [23]. The trainees were divided into healthcare workers, if they were categorised as health or health associate professionals, according to WHO and non-healthcare workers those professions that are involved in disaster response, but don’t pertain to the previously mentioned professional groups [24].

To be included, a learning program must have clear learning objectives and an M&E framework. The objectives should specify measurable outcomes, such as the skills or knowledge participants are expected to acquire [25, 26]. Peer reviewed or not reviewed, empirical or theoretical original research articles written in one of the following languages were included: English, Arabic, French, Romanian, or Italian. Reviews, commentaries, editorials, conference papers, letters to editors, and anecdotal accounts were excluded.

Records that did not address the subject under investigation or for which the full text was not available (directly or made available following contact with the authors) were excluded. Similarly, records were excluded if the courses were addressed to nonprofessional workers, did not present the courses’ learning objectives or did not present the M&E framework.

### Data extraction and analysis

The included records were organized into a Microsoft Excel, Version 2408 (Microsoft Corporation, Redmond, Washington, U.S.) spreadsheet, and the data were extracted, with a focus on course characteristics (course subject, participants, training methodology), learning objectives, and the M&E process (methodology, development process, evaluated items, questions used for evaluation, outcomes, follow-up duration and the fundamental reason for performing M&E). The training topics were categorized following Sarin et al. [27], whereas Anderson et al. revised Bloom’s taxonomy to index the learning objectives because of the strong background and wide usage of the framework [28]. Therefore, the learning objectives were categorized into those addressing the trainees’ ability to “Remember” “Understand”, “Apply”, “Analyse”, “Evaluate” and “Create”. According to the revised taxonomy, “Remembering” involves recalling knowledge from memory, while “Understanding” entails interpreting or making meaning from messages. “Applying” refers to using knowledge in practice, such as through simulations, and “Analysing” requires organizing or differentiating message components. “Evaluating” involves making judgments and offering critiques or recommendations, and “Creating” focuses on producing new knowledge or synthesizing information from learned material [28].

The type of training used was categorized into “classroom-based”, “simulation-based” and “technology-based”, with the simulation types being further classified according to the WHO’s simulation exercise manual [29, 30]. The evaluation methodology was indexed according to the Preparedness and Emergency Response Learning Centre’s framework and Cochrane’s recommendations for data collection [31, 32], whereas the evaluation processes were grouped according to the levels indicated by the New World Kirkpatrick framework [33].

As part of the analysis, the reviewers examined how frequently each evaluation methodology was used for different training topics. The relationships among the learning objectives, categorized according to the revised Bloom’s taxonomy, and the evaluation levels defined by the New World Kirkpatrick framework were also investigated. To visualize these connections and their relative magnitudes, a Sankey plot was used.

## Results

**Fig. 1:**
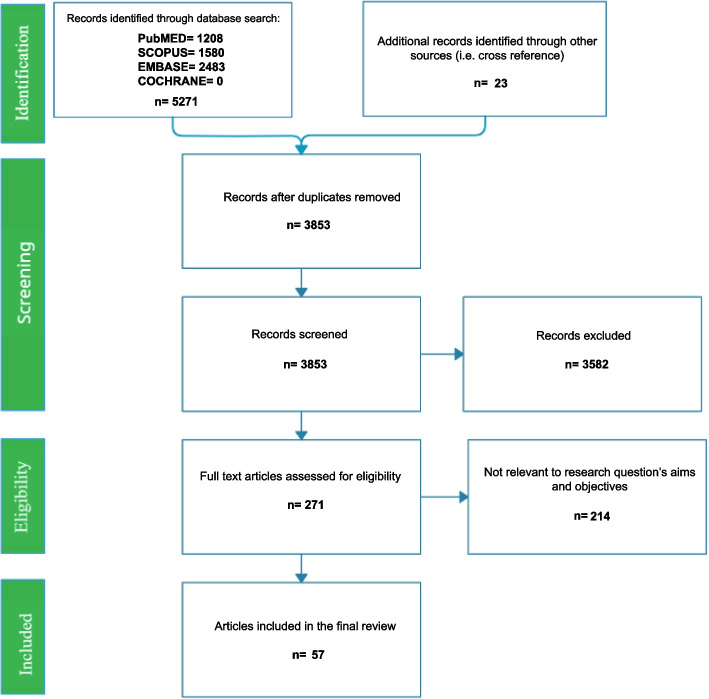
The PRISMA flow diagram [19]

A total of 5,721 articles were identified through the initial database search. An additional 23 records were included through other sources, such as cross-referencing and keyword searches. After removing duplicates, the titles and abstracts of 3,853 records were screened. Of these, 271 were selected for full-text review, resulting in 57 articles that met the established inclusion and exclusion criteria. Detailed information on the article selection process is illustrated in the PRISMA diagram (Fig. 1). A comprehensive summary of the extracted data is provided in Additional File 1.

### Training features

Various healthcare professionals, including undergraduates (*N* = 20), nurses (*N* = 21), medical doctors (*N* = 16), residents (*N* = 6), emergency medical service professionals (*N* = 10), public health workers (*N* = 6), humanitarian professionals (*N* = 1) and pharmacists (*N* = 1), were targeted by the identified DM training programs. Additionally, nonhealthcare professionals such as hospital administrators (*N* = 5), logistics specialists (*N* = 2), government officials (*N* = 2), security workers (*N* = 2), firefighters (*N* = 2), and personnel involved in military programs (*N* = 1) were included in the studies. (Additional File 1).

Classroom-based training was used in 41 trainings, including lectures (*N* = 41), group work (*N* = 17), videos (*N* = 5), case studies (*N* = 4) and serious games (*N* = 1). Technology-based training was used either as blended learning or as a standalone training method by 15 authors. Distance-based online learning (*N* = 14) was used by most of the training organizers, whereas computer-based simulation was reported in 7 trainings and virtual reality simulation in 1. The simulations were used in most of the trainings (*N* = 42), with the main type being drills (*N* = 24), followed by tabletop simulations (*N* = 20), full-scale exercises (*N* = 6) and functional exercises (*N* = 4). The training methodology was not mentioned by 1 article.

The most recurrent training topics were Incident Command System (*N* = 29), Triage (*N* = 22), Communication (*N* = 22), Hospital Disaster Management (*N* = 20) and Public health during Disasters (*N* = 19). Adult teaching, Legal and Ethics, Community disaster preparedness, Safety and Security were present in 3 articles, while Mental health and Health consequences of disasters were taught during 2 trainings.

The trainings were carried out in various economic context ranging from high income countries from North America and Europe to middle income countries from Middle East. (Additional File 1).

### Monitoring and evaluation

**Fig. 2:**
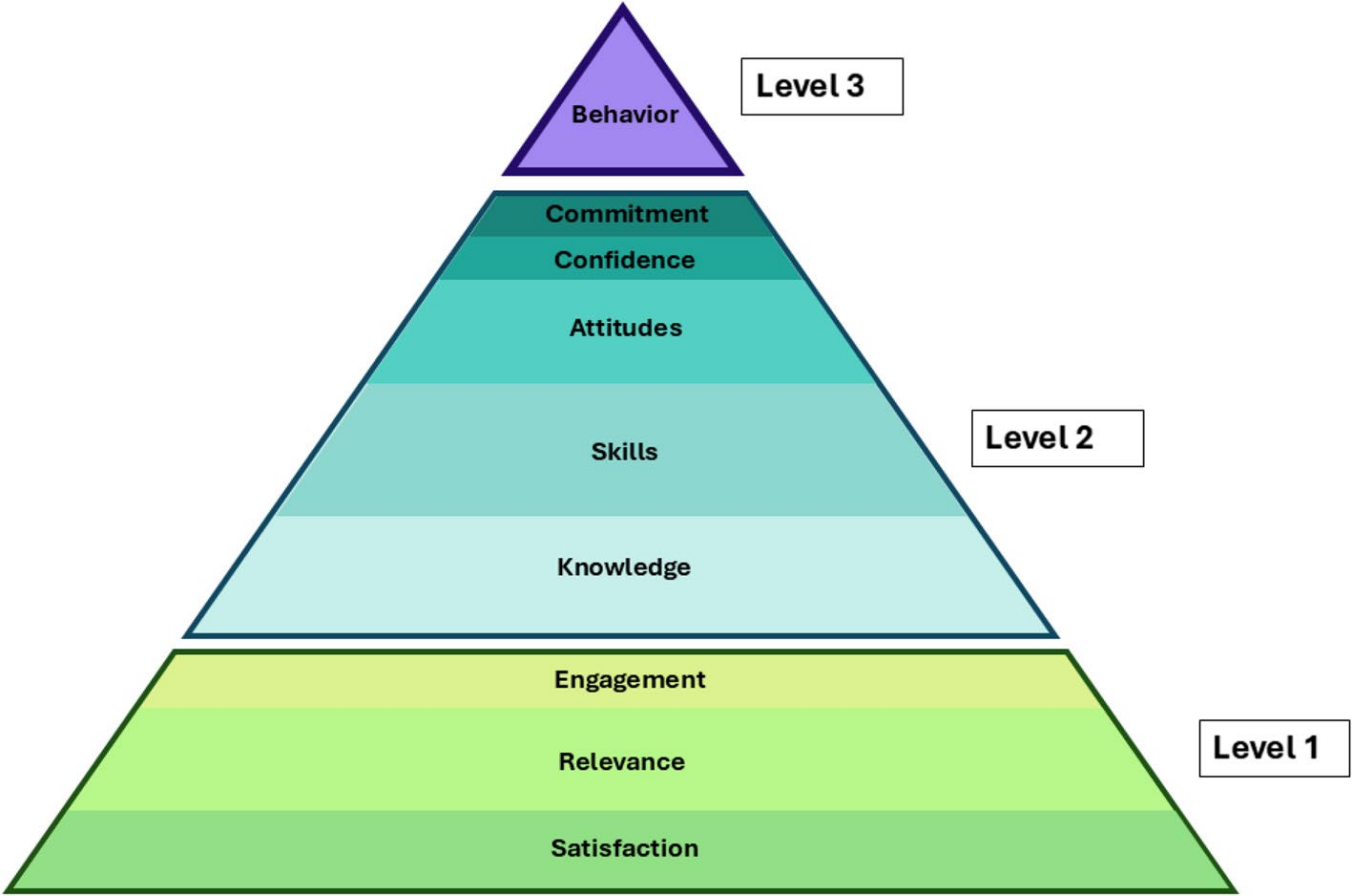
The prevalence of the new world Kirkpatrick levels. Level 1 analyses the Reaction, Level 2 the Learning and Level 3 the Behaviour of the evaluated participants. The height of the bars is representative of the prevalence of each sublevel, the thicker bars signifying a higher prevalent sublevel

Five studies reported the implementation of a monitoring process throughout the course. (Additional File 1) In 2 papers, course organizers reported using questionnaires to monitor the training, whereas interviews were conducted in 2 other studies. Additionally, one author mentioned employing both formal and informal feedback. None of the studies included a validation methodology for their monitoring processes. In contrast, all included articles evaluated the described DM training. According to the New World Kirkpatrick Model, all the studies assessed the learning outcomes of the trainees, most of them evaluating the second level of the model (*N* = 57) (focusing on “Knowledge” (*N* = 50), “Skills” (*N* = 24), and “Attitude” (*N* = 20), compared to “Confidence” (*N* = 15) and “Commitment” (*N* = 4)). The first level was evaluated in 41 articles, with the majority reporting on “Satisfaction” (*N* = 38) and “Relevance” (*N* = 20), whereas “Engagement” was examined in 6 articles. Seven articles investigated the third level of the framework (Fig. 2).

The predominant evaluation method used was the pre-post-test, defined as an assessment of the participants’ knowledge and skills before and after the training (*N* = 41). Deploying a questionnaire either at the beginning or the end of training was a commonly employed method for evaluating training outcomes (*N* = 27). Practical simulations, ranging from tabletops to full-scale exercises, were utilized for evaluation purposes in 7 articles. Conducting interviews with participants and organizing structured discussion groups were reported by 6 studies, while 4 cases utilized formally structured feedback or informal discussions. Finally, observations of participants' behaviours, interactions, and activities during the course, which were based on a predefined set of criteria, were reported by 3 articles. (Additional File 1). However, only one article mentioned using a validated framework for analysing the students’ behaviour, while the other two developed their own set of criteria [34]. None of the articles linked their findings to a behavioural change theory.

A follow-up evaluation, defined as an assessment conducted at a significant interval after the conclusion of the course, was reported by 18 papers. Among them, 3 evaluations were performed within one month after the training ended, 8 between one and six months, 7 between six months and one year and 1 after more than one year, while 1 study did not specify the exact timing of the follow-up evaluation. Notably, theoretical frameworks meant to validate the M&E methodologies were employed by only 16 authors. Pilot studies were described in 10 articles, whereas expert opinion served as the primary validation methodology in 8 cases. Details regarding the frameworks used by the authors can be found in Additional File 1.

### Training topics and their relationships with the evaluation methodology

**Fig. 3:**
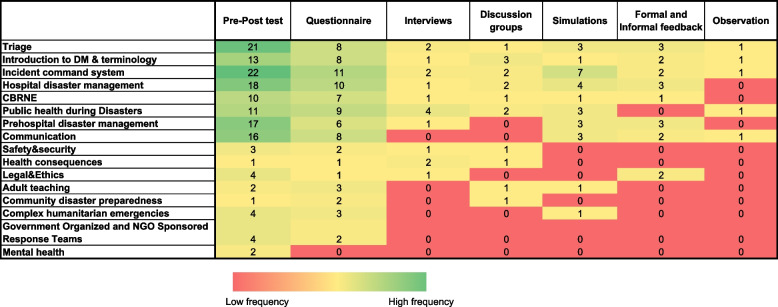
Relationship between the training topics (on the right column) and the evaluation methodologies (on the upper row). The colour palette of the cells corresponds to the frequency of usage of the evaluation methodologies for each training topic. The frequency is represented using a colour range that spans from red to green, where red indicates a low frequency and green signifies the highest frequency

The evaluation methods for different training topics revealed that triage, introduction to disaster management and terminology, and the incident command system were assessed via all available methodologies. Hospital disaster management, prehospital disaster management, chemical, biological, radiological, and nuclear emergencies (CBRNE), communication, and public health during disasters were evaluated via a wide range of methodologies. In contrast, government- and NGO-sponsored response teams and mental health were evaluated via two and one methodologies, respectively (Fig. 3).

### The correlation between learning objectives and M&E

**Fig. 4:**
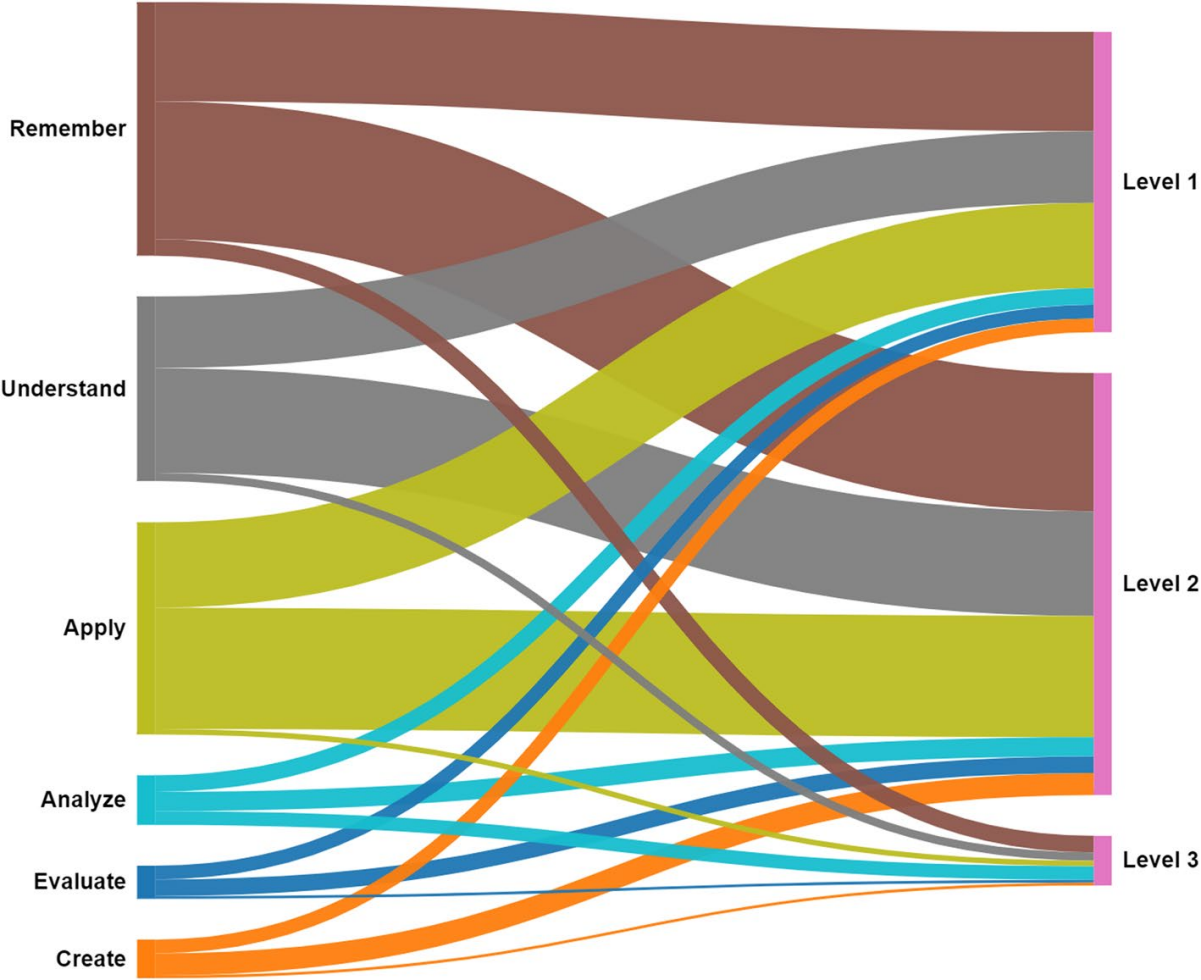
The evaluation levels of the learning objectives. On the left side are the learning objectives stratified according to the revised Bloom’s taxonomy, whereas on the right side, there are the evaluation levels, according to the New World Kirkpatrick framework. Among the evaluation levels, Level 1 analyses the Reaction, Level 2 the Learning and Level 3 the Behaviour of the evaluated participants. The width of each bar represents the number of correlations between the learning objectives and the evaluation levels, with the thicker bars representing a higher correlation

The learning objectives outlined by the studies focused primarily on the "Remember" level of the revised Bloom's taxonomy (*N* = 50), followed by "Apply" (*N* = 44) and "Understand" (*N* = 38) (Fig. 4). Eight trainings were aimed at "Create," 7 at "Analyse," and 6 at "Evaluate". The "Remember" level was assessed primarily via Level 2 of the new Kirkpatrick model (*N* = 50), whereas Level 3 was addressed by 6 studies. The "Apply" objectives were primarily evaluated via Level 2 (*N* = 44) and Level 1 (*N* = 31) objectives of the Kirkpatrick model. Similarly, "Understand" objectives were assessed via Level 2 (*N* = 38) and Level 1 (*N* = 26) objectives, whereas Level 3 objectives were explored in 3 studies. The learning objectives aimed at "Analyse," "Evaluate," and "Create" were investigated primarily via Levell 2 (*N* = 7, *N* = 6, *N* = 8) and Level 1 (N = 6, N = 5, N = 5), with Level 3 being the least addressed (*N* = 2, *N* = 1, *N* = 1).

## Discussion

The present scoping review examined the M&E strategies currently used in DM training. By analysing the main characteristics of DM training, indexing their learning objectives, and studying the M&E processes used, this study highlights the significant diversity in the application of M&E methodologies and frameworks and highlights a mismatch between the stated learning objectives and the M&E process.

In accordance with the literature, there is significant heterogeneity in the typology, topics, and teaching methodologies of DM training, which can be attributed to factors such as the absence of a standardized training protocol and governance in the field [10, 29, 35]. Training participants exhibit a broad spectrum of professional backgrounds, with a notable majority coming from healthcare, highlighting the predominant focus on medical DM over nonmedical sectors [15]. This trend seems to persist despite the literature's call to adopt an interprofessional approach to DM [36, 37]. The training methodologies reported by the studies predominantly favour classical, face-to-face lectures, notwithstanding reports from several authors advocating for the integration of technology into their educational programs. Most of the articles reported the use of simulations, particularly those requiring minimal human or material resources. It is indeed well established that the use of tabletop simulations and drills enhances student engagement and learning while minimizing costs and infrastructure requirements [38–41]. The scarce use of computer-based or virtual reality simulations, although proven to be useful and cost-efficient in the long term as simulation tools, could be explained by the high initial cost and low availability of high-quality, easy-to-use tools [42–44]. Nevertheless, existing evidence suggests that employing multiple learning methods, particularly interactive and simulation-based methods, yields superior results [45–48]. The training topics reported in our study are consistent with those in the literature, emphasizing the competences that are considered fundamental for disaster response [10, 16, 49, 50]. Several after-action reports highlight the communication deficiencies observed during disaster responses [51–55]. Consequently, the strong focus on communication skills in DM training aligns with these observations, paving the way for more effective and efficient soft skills training in the field.

Monitoring was reported by a very small fraction of the studies, which could be explained by their time constraints in delivering the course, but it could also derive from a reporting bias. Moreover, those who did report it omitted a validation methodology, which could be explained by the lack of guidelines in this domain or by the lack of awareness regarding its importance. While running/delivering training, there are several methods for monitoring, ranging from formal or informal feedback, discussion groups, and interviews to questionnaires or technology-based solutions [56, 57]. By not properly monitoring the participants throughout the course, training organizers lack the information needed to improve the programme while it is ongoing. The absence of participants’ immediate feedback or engagement tracking can result in trainees’ disinterest and inefficient learning [58, 59]. Furthermore, without a structured monitoring process, training organizers may fail to identify unmet learning objectives, leading to gaps in knowledge and skills that will need to be filled at a later time [60].

Given the urgency and unpredictability of disasters, it is crucial to have responders continuously trained and prepared to integrate their knowledge into practice, making training evaluation of paramount importance. The prevalent use of the second level of the New World Kirkpatrick Model in the evaluation of all included studies underscores the emphasis the training organizers put on assessing skills and knowledge. This level is typically measured via nonparticipatory methods, such as multiple-choice questions or skill tests, which do not provide an explanation of the learning outcome. Additionally, focusing predominantly on skills and knowledge acquisition predisposes trainees to gaps in understanding whether they can effectively apply what they have learned or if the acquired knowledge will translate into actual competencies and influence their behaviour during disaster response [61, 62]. Moreover, without objectively assessing students’ ability to apply newly acquired knowledge and skills, the included papers focus on self-reported confidence and reported skills, which are widely recognized as not being correlated with actual real-life behaviour [63–65].

The limited number of articles evaluating behavioural change following the training activity highlights a significant drawback in the M&E of the DM training programmes, findings that are in accordance with the literature [11, 66, 67]. Existing risk communication studies have consistently shown a weak correlation between being knowledgeable about preparedness strategies and translating that knowledge into actual preparedness actions [68–70]. Hence, without assessing behavioural changes, training organizers cannot ascertain the training's impact on participants' DM response performance, leading to uncertainty regarding the effectiveness of the capacity-building programme. One reason that could explain this finding is the absence of validated frameworks tailored to the specific needs of DM training. Measuring behavioural changes during an actual disaster response is indeed challenging owing to the multitude of factors influencing response efforts, compounded by the infrequency of disasters [66]. Another possible reason is the lack of practical guidance on how to adapt the existing methods of evaluation to the particularities of the DM.

A small number of studies reported a longitudinal assessment of the participants after the training’s completion, with the majority reassessing trainees within the first 6 months post training. These findings align with previous research underscoring the importance of longitudinal evaluation to comprehensively grasp the complexity involved in the learning process, the transfer of knowledge into practice, and the long-term effects of training [71].

The evaluation methodologies employed, such as pre-post-tests and questionnaires, are well documented in the literature as being widely used owing to their quantitative, measurable and comparable nature, along with their comfortable usage, on the basis of the trainers’ expertise with the tool [43, 72, 73]. However, these methods have several limitations, including their inability to collect qualitative data on changes in students' attitudes and their susceptibility to biases, such as response shifts, practice effects, and spontaneous improvement [72, 74, 75].

Formal and informal feedback, alongside qualitative evaluation, were identified in a restricted number of academic papers. There is compelling evidence indicating that these methods of data collection can greatly aid educators in conducting a more comprehensive assessment of the course's efficacy and efficiency, thus equipping them with the invaluable insights required to improve the course [76, 77].

Despite evidence from the literature that standardized protocols of M&E enhance training quality and improve participant outcomes [78], only a limited number of studies have employed established, validated frameworks or pilot studies for their M&E processes, potentially contributing to the observed variability among reported methodologies. The absence of a framework tailored to DM characteristics and a limited awareness of the subject may also contribute to this situation [13].

The different methodologies used to assess prevalent and recurring subjects in DM stress the importance of expertise and familiarity with frequently studied subjects compared with less frequent ones. Employing multiple evaluation approaches for the same subject enables instructors to gain a comprehensive understanding of the learning process, thereby facilitating the formulation of pertinent conclusions regarding course outcomes. Triage and incident command systems, for example, are extensively documented in the literature, featuring validated key performance indicators and numerous concrete evaluation examples [79–82].

However, several less common topics were assessed via a limited range of methods, primarily pre-post-tests and questionnaires. For example, evaluating mental health training with these methodologies might prove ineffective due to the complexity of interpersonal skills and the practical nature of mental health interventions, aspects that are inadequately captured by solely quantitative measurement tools [83]. Similarly, training programs for government and NGO-sponsored response teams represent a relatively new concept that is increasingly becoming professionally managed and standardized, with significant efforts from the WHO to support their development [84]. These teams have unique characteristics, including diverse team member backgrounds, limited prior collaboration, and varied professional experiences, all of which complicate the implementation of M&E. This complexity may explain the limited use of diverse evaluation methodologies in assessing these programs [36, 85, 86].

When the learning objectives declared by the authors are analysed and categorized according to the revised Bloom's taxonomy, it is obvious that most of the objectives are focused on trainees’ ability to “Remember”, followed by the ability to “Apply” and “Understand”. The small proportion of the training organizers that aimed at improving the trainees’ ability to “Create”, “Analyse” and “Evaluate”, which are the core of developing critical thinking skills, highlights the need to integrate these goals in future training.

Furthermore, the correlation between the revised Bloom's taxonomy levels (learning objectives) and the New World Kirkpatrick levels (evaluation methodology) reveals that a significant number of the articles have assessed trainees' skills (ability to "Apply" and "Create") using the second level of the New World Kirkpatrick framework as opposed to the third level. The second level of the New World Kirkpatrick Model aims at evaluating trainees’ skills and knowledge by the end of the course, documenting whether they have learned the concepts that were taught. In contrast with the third level, the second level does not analyse whether the students are able to apply or integrate the newly developed skills in their day-to-day life.

Moreover, a low number of studies have evaluated course effectiveness via simulations. While evaluating participants' performance through simulations can have merits, it is important to note that simulations do not accurately reflect behavioural change and are not necessarily comparable to real-life improvements, as suggested by existing evidence [62]. The literature encourages the evaluation of behavioural change (Level 3 of the New World Kirkpatrick Model) to ensure knowledge and skill transfer to the job, with a wide variety of tools being available [87, 88]. By not analysing trainees’ behavioural changes and the impact of training on their day-to-day work, the correlation between acquired skills and knowledge and their impact on real-life DM is unlikely to be certain. In addition, dangerous conclusions could be drawn from a deceptive evaluation, given that some of the included articles consider their graduates to be ready to deploy, following the course conclusion. Therefore, the trainees might face hardship when dealing with real-life situations, although the post training evaluation of their skills or knowledge was satisfactory.

This mismatch between the learning objectives and the evaluated outcomes has the potential to lead to unreliable evaluation outcomes, possibly impacting patients.

While documenting this review, the authors observed a gap in the literature and frameworks on linking the learning objectives with the M&E strategies in DM capacity-building programs. Without clear learning objectives and a strong correlation between them and the M&E strategy, false conclusions could be drawn while analysing the outcomes of training. Future research is needed to fill this scientific gap.

### Limitations

There are two main limitations of the current review. First, despite screening multiple databases, it is important to note that PubMed and EMBASE primarily consist of medical and pharmaceutical content. Therefore, it is understandable that training topics and participants predominantly pertain to the healthcare sector. Second, an additional limitation was the inability to establish a direct correlation between each learning objective and its respective evaluation criteria. When constructing Figure nr. 4, the entire set of learning objectives was correlated with the complete evaluation protocol for each study instead of correlating each objective with its corresponding evaluation criteria.

## Supplementary Information

Supplementary Material 1. [89–163].

## Data Availability

Data is provided within the manuscript or supplementary information files.
