# Supplementary material for: Monitoring and evaluation in disaster management courses: a scoping review
Source: BMC Med Educ. 2025 Feb 6;25:188. doi: 10.1186/s12909-025-06659-0 (PMC11800627; doi:10.1186/s12909-025-06659-0)
Supplement: Supplementary file 1 — Supplementary Material 1. [89–163]. [file 12909_2025_6659_MOESM1_ESM.docx]

| **Author and publication year** | **DOI** | **Country** | **Training Topic** | **Participants** | **Type of training** | **Learning objectives (Revised Bloom's Taxonomy)** | **Evaluation instruments** | **Evaluated items (New World Kirkpatrick)** | **Evaluation's validation methodology & Framework [REF]** | **Follow-up evaluation** | **Monitoring instruments** |
| --- | --- | --- | --- | --- | --- | --- | --- | --- | --- | --- | --- |
| Alan, H. et al., 2023 [89] | 10.1017/dmp.2023.8 | Türkiye | Introduction, Incident Management System | Nurses | Technology (Elearning) | Recalling, Exemplifying | Pre-Post Tests | Level 2(Knowledge) | Expert opinion | - | - |
| Al-Qbelat, R.M.; Subih, M.M.; Malak, M.Z., 2022 [90] | 10.1177/00469580221130881 | Jordan | Introduction, Triage, Prehospital, CBRNE, Mental Health | Nurses | Classroom (Lectures, Groupwork, Video) | Executing, Recognizing, Recalling, Explaining, | Pre-Post Tests | Level 2 ( Knowledge, Skill, Attitude, Confidence) | Pilot study, Theoretical Framework/Model [91] | - | - |
| Altillo, B.S.A. et al., 2021 [92] | 10.1186/s12909-021-02616-9 | United States of America | Health Consequences, Public Health | Students | Technology (Elearning) | Recalling, interpreting, Differentiating, | Questionnaires, Interviews | Level 1(Satisfaction, Relevance), Level 2(Knowledge, Confidence) | - | - | - |
| Back, D.A. et al., 2019 [93] | 10.1093/milmed/usy250 | Germany | Triage, MCI, Management, CBRNE | Students | Technology (Elearning, Computer simulation), Classroom(Groupwork, Case study, Lectures), Simulation (Drill) | Recalling, Recognizing, Executing, Explaining | Pre-Post Tests | Level 1(Satisfaction), Level 2(Knowledge) | - | - | - |
| Bajow, N. et al., 2022 [94] | 10.1186/s12909-022-03427-2 | Saudi Arabia | Hospital, CBRNE, Triage, Satefy&Security, Health Consequences | Medical doctors, Nurses | Simulation (Tabletop exercises, Functional exercise), Technology (Computer exercise, Elearning), Classroom (Lectures) | Recalling, Recognizing, Executing, | Pre-Post Tests, Discussion Groups, Informal Feedback | Level 2(Knowledge, Skill, Attitude), Level 1(Satisfaction, Relevance), Level 3 | Theoretical Framework\Model [95] | 12 months | - |
| Bajow, N.A.; Alassaf, W.I.; Cluntun, A.A., 2018 [96] | 10.1017/S1049023X18000791 | Saudi Arabia | Introduction, Triage, Incident Management System, MCI, Legal&Ethics, Communication | Nurses, Medical doctors, Emergency Medical Service professionals, Pharmacists | Classroom (Lectures, Groupwork), Simulation (Tabletop exercises, Drill) | Recalling, Classifying, Comparing, Interpreting, Explaining, Executing, Differentiating | Pre-Post Tests, Feedback Form (?) | Level 1(Satisfaction, Relevance), Level 2(Knowledge, Confidence) | - | 8 months | - |
| Bajow, N.A.; Alawad, Y.I.; Aloraifi, S.M., 2019 [97] | 10.1017/S1049023X19004977 | Saudi Arabia | Complex Humanitarian Emergencies | - | Classroom (Lectures, Groupwork, Case study), Simulation (Tabletop exercises, Drills) | Recalling, Executing | Pre-Post Test, Questionnaire | Level 1(Satisfaction), Level 2(Knowledge, Skill) | Theoretical Framework\Model [98] | 10 months | - |
| Bank, I.; Khalil, E., 2016 [99] | 10.1017/S1049023X16000704 | Canada | Triage | Medical doctors, Residents | Classroom (Lectures), Simulation (Drill) | Recognize, Executing, Implementing, Comparing, | Pre-Post Test, Questionnaire | Level 1(Satisfaction), Level 2(Skill, Knowledge) | - | 6 months | - |
| Beaton, R.D.; Johnson, L.C., 2002 [100] | 10.1017/S1049023X00000339 | United States of America | Public Health | Emergency Medical Service professionals, Firefighters | Classroom (Lectures) | Recalling, recognizing, executing, Implementing | Pre-Post Tests | Level 1(Satisfaction), Level 2(Confidence, Knowledge) | Pilot study | 4 months | - |
| Bentley, S. et al., 2019 [101] | 10.15766/mep_2374-8265.10823 | United States of America | Triage, Hospital, MCI Management | Residents, Nurses, Hospital administrators | Simulation (Drill) | Executing, Explaining | Questionnaire, Simulation | Level 1 (Satisfaction, Relevance), Level 2(Knowledge, Skill) | - | - | - |
| Bodas, M. et al., 2022 [102] | 10.1017/dmp.2020.359 | Israel | Prehospital, Hospital, Incident Management System, Communication, Complex Humanitarian Emergencies, Ngo Sponsored Response Team | Medical doctors, Nurses, Emergency Medical Service professionals, Logisticians | Simulation (Tabletop exercises, Functional Exercise) | Interpreting, Executing, Recognizing, Generating, Organizing, Attributing | Pre-Post Test, Questionnaire | Level 1 (Satisfaction, Engagement, Relevance), Level 2(Knowledge, Attitude, Skill) | Theoretical Framework\Model [103,104] | - | - |
| Carenzo, L. et al., 2022 [105] | 10.1017/dmp.2022.84 | Italy | MCI Management, Triage | Emergency Medical Service professionals, Medical doctors, Nurses | Technology (Elearning, Computer simulation), Simulation (Tabletop exercises), Classroom (Lectures) | Recalling, Executing | Pre-Post Test, Questionnaire | Level 1(Satisfaction, Relevance), Level 2(Knowledge) | Theoretical Framework\Model [106,107] | - | - |
| Chang, C.-W. et al., 2022 [108] | 10.1016/j.nedt.2022.105613 | Taiwan | Incident Management System, Triage, Prehospital | Nurses | Technology (Virtual reality simulation) | Recognize, Inferring, Executing, Implementing | Pre-Post Tests | Level 2(Confidence) | Theoretical Framework\Model, Expert opinion [109,110] | 1 month | - |
| Chung, S. et al., 2018 [111] | 10.1017/dmp.2017.137 | United States of America | Public Health | Medical doctors | Simulation (Tabletop exercises) | Planning, Critiquing, Interpreting | Questionnaire | Level 2(Knowledge, Confidence, Attitude) | - | 6 months | - |
| Collander, B. et al., 2008 [112] | 10.1017/s1049023x00005598 | United States of America | Incident Management System, Communication | Medical doctors, Nurses, Emergency Medical Service professionals, Hospital administrators, Security technicians | Classroom (Lectures), Simulation (Tabletop exercises, Drill) | Recognizing, Executing, Interpreting, Inferring | Pre-Post Test, Questionnaire | Level 1(Relevance), Level 2(Knowledge, Attitude) | - | - | - |
| Cranmer, H. et al., 2014 [113] | 10.1017/S1049023X13009217 | United States of America | Complex Humanitarian Emergencies, Safety&Secturity | Humanitarian Professionals | Simulation (Full scale exercise) | Executing, Generating | Questionnaire | Level 2(Skill, Knowledge) | - | 6 months | - |
| Daniel, P. et al., 2016 [114] | 10.1017/S1049023X16000212 | United States of America | Hospital, CBRNE, ICS, Adult Teaching | Residents | Simulation (Drill) | Recalling, Executing, Implementing | Pre-Post Test, Questionnaire, Simulation | Level 2 (Knowledge, Skill, Attitude) | - | - | - |
| Dastyar, N.; Nazari, M.; Rafati, F., 2023 [115] | 10.1017/dmp.2022.269 | Iran | Introduction, Triage, Prehospital, Legal&Ethics | Students | Technology (Elearning), Simulation (Drill) | Recalling, Executing | Pre-Post Tests, Interviews | Level 1(Satisfaction), Level 2(Knowledge, Skill, Attitude) | Theoretical Framework\Model, Expert opinion [116,117] | 2 months | - |
| Farhat, H. et al., 2022 [118] | 10.5339/jemtac.2022.38 | Qatar | CBRNE, MCI, Prehospital Disaster Management, Hospital Disaster Mitigation, Preparedness, Response And Recovery | Medical doctors, Emergency Medical Service professionals, Nurses | Classroom (Lectures, Groupwork, Video), Simulation (Tabletop exercises) | Classifying, Inferring, Recognizing, Explaining | Pre-Post Test, Questionnaire | Level 1(Satisfaction), Level 2( Knowledge, Skill) | - | - | - |
| Franc, J.M.; Nichols, D.; Dong, S.L., 2012 [119] | 10.1017/S1049023X11006807 | Canada | Introduction, Triage, Incident Management System, Hospital Disaster Mitigation, Preparedness, Response And Recovery | Residents | Classroom (lectures), Simulation (Tabletop exercises) | Recalling, Executing, Implementing, Generating, Planning | Pre-Post Test, Questionnaire | Level 2 (Confidence, Attitude, Knowledge, Skill) | Theoretical Framework\Model, Pilot study [120] | - | - |
| Gershon, R.R.M. et al., 2009 [121] | 10.1017/S1049023X00007421 | United States of America | Public Health | Emergency Medical Service professionals | Classroom (Lectures), Simulation (Drill) | Recalling, Executing, Inferring | Pre-Post Test, Questionnaire | Level 2 (Knowledge, Commitment) | Expert opinion | - | - |
| Ghiga, I. et al., 2021 [122] | 10.1016/j.vaccine.2020.11.047 | United States of America | Introduction, Public Health | - | Simulation (Tabletop exercises) | Interpreting, Inferring, Explaining, Recalling, Recognizing, Executing, Planning, Checking | Questionnaire | Level 1 (Satisfaction, Relevance, Engagement), Level 2 (Knowledge) | Pilot study | - | - |
| Glow, S.D. et al., 2013 [123] | 10.1017/S1049023X13000423 | United States of America | Triage, MCI Management, Communication | Nurses, Medical doctors, Hospital administrators, Firefighters | Simulation (Functional exercise), Classroom (Lectures) | Recognizing, Executing, Exemplifying, Summarizing | Pre-Post Tests, Simulation | Level 2 (Knowledge) | - | - | - |
| Heaslip, G.; Stuns, K.-K., 2019 [34] | 10.1108/JHLSCM-12-2018-0080 | Finland | Incident Management System | Medical doctors, Nurses | Classroom (lectures), Simulation (Drill) | Recognizing, Recalling, Interpreting, Exemplifying | Questionnaire, Observation, Interviews | Level 1 (Satisfaction), Level 2, Level 3 | Theoretical Framework\Model [95] | - | Questionnaire |
| Henze, S.M. et al., 2022 [124] | 10.1186/s12909-022-03783-z | Germany | Introduction, Incident Management System, Triage, Prehospital, Hospital, Public Health | Students | Technology (Elearning, Computer simulation) | Recalling, Executing, Exemplifying, Explaining | Pre-Post Tests | Level 1(Satisfaction, Engagement), Level 2(Attitude) | Expert opinion | - | Questionnaire |
| Hermann, S. et al., 2021 [125] | 10.1186/s12909-021-03043-6 | Germany | Introduction, Triage, Prehospital, Hospital, CBRNE, Legal&Ethics | Students | Technology (Elearning, Computer simulation), Simulation (Tabletop exercises, Full scale exercise), Classroom(Lectures) | Explaining, Executing, Recalling | Pre-Post Tests, Questionnaire, Informal Feedback | Level 1(Satisfaction), Level 2(Knowledge) | - | - | - |
| Horney, J.A., 2009 [126] | 10.1177/003335490912400421 | United States of America | Public Health, Community Disaster Preparedness | Government officials | Technology (Elearning) | Recalling | Questionnaire | Level 1(Satisfaction), Level 2(Attitude, Skill), Level 3 | Pilot study | 6 months | - |
| Hsu, C.-C. et al., 2022 [127] | 10.6705/j.jacme.202212_12(4).0003 | Taiwan | Triage, Prehospital, CBRNE | Students | Technology (Elearning), Classroom (Lectures, Groupwork), Simulation (Drill, Functional Exercises) | Recalling, Executing | Pre-Post Tests | Level 1 (Satisfaction), Level 2(Knowledge, Skill) | - | - | - |
| Kaim, A. et al., 2023 [86] | 10.3389/fpubh.2023.1150030 | Portugal, Germany, Norway, and Türkiye | Prehospital, Hospital, Communication, Safety&Security, Government Response Teams, Ethical, Mental Health, Complex Humanitarian Emergencies | Nurses, Residents, Emergency Medical Service professionals, Logisticians, Hospital administrators | Simulation (Drill) , Technology (Elearning), Classroom (Lectures, Groupwork) | Recognizing, Executing, Inferring, Generating, Checking, Organizing, Implementing, Critiquing, Interpreting, Planning, Attributing | Pre-Post Tests | Level 1 (Satisfaction, Relevance), Level 2(Knowledge, Skill, Confidence) | Pilot study, Theoretical Framework/Model, Expert opinion[128] | - | - |
| Kaji, A.H.; Coates, W.; Fung, C.-C., 2010 [129] | 10.1080/10401331003656561 | United States of America | Introduction, Incident Management System, Hospital Disaster Mitigation, Preparedness, Response And Recovery | Students | Classroom (Lectures, Drill observation) | Recalling, Inferring, Explaining | Questionnaire, Discussion Group, | Level 1(Relevance, Engagement), Level 2(Skill, Attitude) | - | - | - |
| Kaplan, B.G. et al., 2012 [130] | 10.1111/j.1525-1446.2011.00960.x | United States of America | Public Health, Incident Management System, Communication | Students | Classroom (Lectures), Simulation (Drill) | Recalling, Recognizing, Critiquing, Executing | Pre-Post Tests, Simulation | Level 1(Satisfaction, Relevance), Level 2(Confidence, Knowledge) | - | - | - |
| Kennedy, B.; Carson, D.S.; Garr, D., 2009 [131] | 10.1097/01.PHH.0000345980.49798.d2 | United States of America | Public Health | Public health workers | Classroom (Lectures) | Recognizing, Executing, Implementing | Pre-Post Tests | Level 1(Satisfaction, Relevance), Level 2(Knowledge) | Theoretical Framework\Model [132] | 12 months | - |
| Kesler, S. et al., 2022 [133] | 10.1017/dmp.2022.28 | United States of America | Introduction | Social workers, Security technicians, Government officials | Simulation (Full scale exercise), Classroom (Lectures) | Executing, Implementing, Exemplifying, Recognizing, Recalling | Pre-Post Test, Questionnaire | Level 1(Satisfaction), Level 2(Knowledge) | - | - | - |
| Lennquist Montan, K. et al., 2014 [134] | 10.1007/s00068-013-0350-y | Sweden | Incident Management System, Communication, Triage | Medical doctors, Nurses, Emergency Medical Service professionals, Military personnel, Hospital administrators | Classroom (Lectures), Simulation (Tabletop exercises) | Recognizing, Executing | Pre-Post Tests | Level 2(Knowledge, Attitude, Skill) | - | - | - |
| Levoy, K.; DeBastiani, S.D.; McCabe, B.E., 2018 [135] | 10.1017/dmp.2017.150 | United States of America | Introduction, Incident Management System | Students | Simulation (Tabletop exercises, Drill, Full scale exercise), Classroom (Lectures), Technology (Elearning) | Recalling, Summarizing, Exemplifying, Executin | Pre-Post Tests, Simulation | Level 2(Knowledge, Confidence) | - | - | - |
| Montana, M. et al., 2019 [136] | 10.3352/jeehp.2019.16.19 | France | Introduction, CBRNE | Students | Classroom (Lectures) | Recognising, Exemplifying, Inferring, Explaining, Executing | Questionnaire | Level 1(Satisfaction), Level 2(Knowledge) | Expert opinion | 24 months | - |
| Morrison, A.M.; Catanzaro, A.M., 2010 [137] | 10.1111/j.1525-1446.2010.00838.x | United States of America | Public Health | Students | Classroom (Lectures, Video), Simulation (Drill) | Recognizing, Executing, Implementing | Questionnaire, Observation | Level 1 (Satisfaction, Relevance, Engagement), Level 2(Knowledge, Skill, Attitude) | Theoretical Framework\Model [138] | - | - |
| Nybo, S.E.; Klepser, S.A.; Klepser, M., 2020 [139] | 10.1016/j.cptl.2020.01.037 | United States of America | CBRNE | Students | Classroom (Lectures, Video, Serious games) | Interpreting, Exemplifying, Planning, Explaining | Questionnaire | Level 1 (Satisfaction, Relevance), Level 2(Knowledge, Skill) | - | - | - |
| Pate, A. et al., 2016 [140] | 10.5688/ajpe80350 | United States of America | Introduction, Public Health | Students | Simulation (Tabletop exercises) | Recalling, Interpreting, Executing | Pre-Post Tests, Discussion Groups | Level 1(Satisfaction), Level 2 (Knowledge, Attitude, Confidence, Commitment) | - | - | - |
| Pesiridis, T. et al., 2015 [141] | 10.1016/j.nepr.2014.02.001 | Greece | Hospital Disaster Mitigation, Preparedness, Response | Nurses | Classroom (Case study, Lectures, Group work), Simulation (Drill) | Recalling, recognizing, Executing | Pre-Post Tests | Level 2(Knowledge, Attitude) | Theoretical Framework\Model, Pilot study [142,143] | 5 months | - |
| Pitts, J. et al., 2009 [144] | 10.1080/14739879.2009.11493816 | United Kingdom | Public Health | Medical doctors | Classroom (Lectures, Groupwork, Video) | Recognizing, Inferring | Interviews | Level 1 (Satisfaction, Engagement, Relevance), Level 2(Knowledge, Confidence, Attitude) | - | 1 month | - |
| Pryor, E. et al., 2006 [145] | 10.1017/S1049023X00003289 | United States of America | Community Disaster Preparedness, Public Health, CBRNE, Hospital Disaster Mitigation, Preparedness, Response And Recovery | Nurses, Medical doctors, Emergency Medical Service professionals | Simulation (Tabletop exercises) | Differentiating, Recalling, Exemplifying, Recognizing, Executing, Generating, Implementing, Planning, Comparing, Checking, Critiquing | Pre-Post Test, Questionnaire | Level 1 (Satisfaction, Relevance), Level 2(Knowledge, Skill), Level 3 | Theoretical Framework\Model, Expert opinion [146] | - | Formal and informal feedback |
| Qureshi, Kristine A. et al., 2004 [147] | 10.1097/00003727-200407000-00011 | United States of America | Public Health, Government Organized Response Teams, | Nurses | Classroom (Lectures) | Recognizing, Explaining, Interpreting, Exemplifying | Pre-Post Tests | Level 2(Attitude, Knowledge, Commitment) | Pilot study | 1 month | - |
| Ragazzoni, L. et al., 2020 [148] | 10.1097/MEJ.0000000000000668 | Italy | Introduction, Adult Teaching | Students | Simulation (Tabletop exercises), Technology (Computer exercise, Elearning), Classroom (Lectures) | Recalling, Implementing | Pre-Post Test, Questionnaire | Level 1 (Satisfaction), Level 2(Knowledge, Skill) | - | - | - |
| Ripoll-Gallardo, A. et al., 2020 [149] | 10.1186/s13049-020-00778-x | Italy | MCI, Triage, Incident Management System, Public Health, Complex Humanitarian Emergencies | Residents | Technology (Elearning, Computer simulation), Classroom (Lectures, Groupwork), Simulation (Tabletop exercises, Full scale exercise) | Interpreting, Exemplifying, Explaining, Differentiating, Executing, Implementing | Pre-Post Tests, Simulation | Level 1, Level 2, Level 3 | Theoretical Framework\Model [95] | - | - |
| Sarpy, S.A. et al., 2005 [150] | 10.1097/00124784-200511001-00013 | United States of America | Public Health, Incident Management System, Communication | Public health workers | Classroom(Lectures), Simulation (Tabletop exercises) | Recalling, Interpreting, Recognising, Executing | Pre-Post Tests, Simulation | Level 1 (Satisfaction, Relevance),Level 2(Confidence, Knowledge) | Theoretical Framework\Model [151,152] | - | - |
| Scott, L.A. et al., 2012 [153] | 10.5055/ajdm.2012.0093 | United States of America | Introduction, Incident Management System, Communication, Triage | Students, Medical doctors, Nurses | Classroom (Lectures, Groupwork), Simulation (Drill) | Recognizing, Recalling, Differentiating, Interpreting, Inferring, Summarizing, Executing | Pre-Post Tests, Observation | Level 2(Knowledge, Skill, Confidence) | - | 6 months | - |
| Scott, L.A. et al., 2013 [154] | 10.1017/S1049023X13000368 | United States of America | Introduction, Incident Management System, Triage, Communication, Safety&Security | Students, Medical doctors, Nurses | Classroom (Lecture, Groupwork), Simulation (Tabletop exercises, Drill) | Recalling, Exemplifying, Interpreting, Comparing, Executing, Summarizing | Pre-Post Test, Questionnaire | Level 1 (Satisfaction), Level 2(Skill, Knowledge, Attitude) | - | - | - |
| Scott, L.A. et al., 2018 [155] | 10.1017/dmp.2017.30 | United States of America | Incident Management System, Triage, Communication | Students, Medical doctors, Nurses | Classroom(Lectures, Groupwork), Simulation (Functional Exercise) | Recalling, Exemplifying, Interpreting, Comparing, Executing, Summarizing | Pre-Post Tests | Level 2(Skill, Knowledge) | - | - | - |
| Silenas, R. et al., 2008 [156] | 10.1080/10401330701798311 | United States of America | Incident Management System, MCI, Communication | Students | Classroom (Lectures, Groupwork), Simulation (Tabletop exercises) | Recalling, Recognizing, Interpreting, Explaining | Pre-Post Tests, Informal Feedback | Level 1(Satisfaction), Level 2(Knowledge, Attitude) | - | - | - |
| Tower, C. et al., 2016 [157] | 10.1017/dmp.2016.11 | United States of America | Community Disaster Preparedness, Introduction | Public health workers | Classroom(Lectures) | Recalling, Exemplifying, Comparing, Explaining | Discussion Group | Level 1 (Satisfaction), Level 2( Confidence, Commitment) | - | - | - |
| Tsai, Y.-D. et al., 2020 [158] | 10.1097/MD.0000000000020230 | Taiwan | MCI, Triage, Prehospital, Hospital, Incident Management System, CBRNE | Students | Classroom(Lectures, Groupwork), Simulation (Drill) | Recalling, Executing, Implementing | Pre-Post Test, Questionnaire | Level 1(Satisfaction, Relevance), Level 2 (Knowledge) | - | - | - |
| Wang, C. et al., 2008a [159] | 10.1186/1471-2458-8-377 | Popular Republic of China | Public Health | Public health workers | Simulation (Drill), Classroom (Groupwork, Lectures) | Recalling, Recognizing, Interpreting, Exemplifying, Critiquing, Implementing | Questionnaires, Interviews | Level 1(Satisfaction, Relevance), Level 2(Knowledge) | Pilot study | 12 months | Interviews |
| Wang, C. et al., 2008b [160] | 10.1016/j.puhe.2007.08.006 | Popular Republic of China | Public Health, Government Organized Response Teams, Communication | Public health workers | Classroom (Groupwork, Case study), Simulation (Drill) | Recalling, Recognizing, Exemplifying | Pre-Post Test, Questionnaire | Level 2(Knowledge, Skill, Attitude) | Pilot study | 12 months | Interviews |
| Wetta-Hall, R. et al., 2006 [161] | 10.3928/00220124-20060301-03 | United States of America | Public Health, Incident Management System | Nurses | - | Recognizing, Executing, Implementing | Discussion Group, Interview | Level 1(Satisfaction, Relevance), Level 2(Knowledge), Level 3 | - | - | - |
| Wiesner, L. et al., 2018 [162] | 10.1016/j.jemermed.2017.12.008 | United States of America | CBRNE, Prehospital, Hospital | Students | Classroom(Lectures), Simulation (Drill) | Recalling, Interpreting, Executing, Exemplifying | Pre-Post Tests | Level 2(Knowledge) | - | - | - |
| Wright, Kate S. et al., 2010 [163] | 10.1177/00333549101250S515 | United States of America | Adult Teaching | Public health workers | Classroom(Lectures), Simulation (Drill, Full scale exercise) | Recalling, Implementing | Questionnaire, Discussion Group | Level 1(Satisfaction), Level 2(Knowledge), Level 3 | - | 12 months | - |

Abbreviations: MCI= mass casualty incident; CBRNE= Chemical, Biological, Radiological, Nuclear, and high yield Explosives
